# Supplementary material for: Anti-Inflammatory Effects of Mytilus coruscus Polysaccharide on RAW264.7 Cells and DSS-Induced Colitis in Mice
Source: Mar Drugs. 2021 Aug 20;19(8):468. doi: 10.3390/md19080468 (PMC8400803; doi:10.3390/md19080468)
Supplement: Supplementary file 1 [file marinedrugs-19-00468-s001.zip › marinedrugs-1333790-supplementary.pdf]

Appendix A1

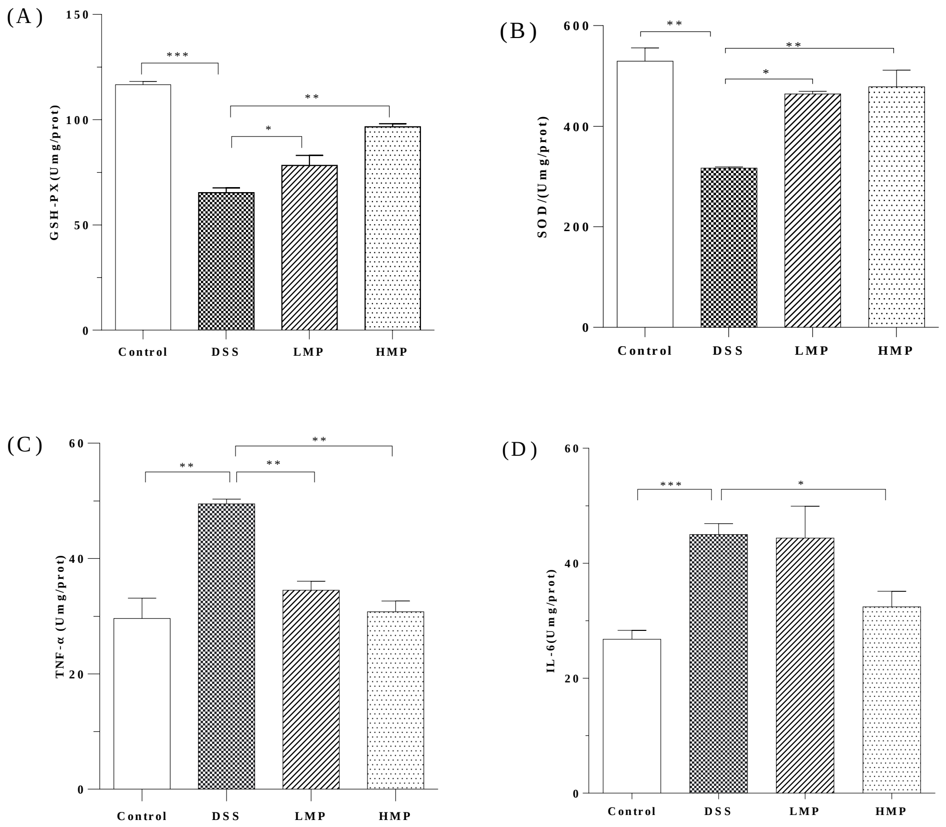

Scheme 1. Effect of MP on intestinal oxidative stress (GSH-Px (**A**) and SOD (**B**)), and inflammatory response (TNF-*α* (**C**) and IL-6 (D)) in DSS-induced colitis mice. The data are presented as means ± SEM. Significant differences in the results are analyzed by 1-way ANOVA analysis with post hoc independent samples *t*-tests (*p* values <0.05, <0.01, and <0.001 are indicated by *, **, and ***, respectively).

Appendix A2

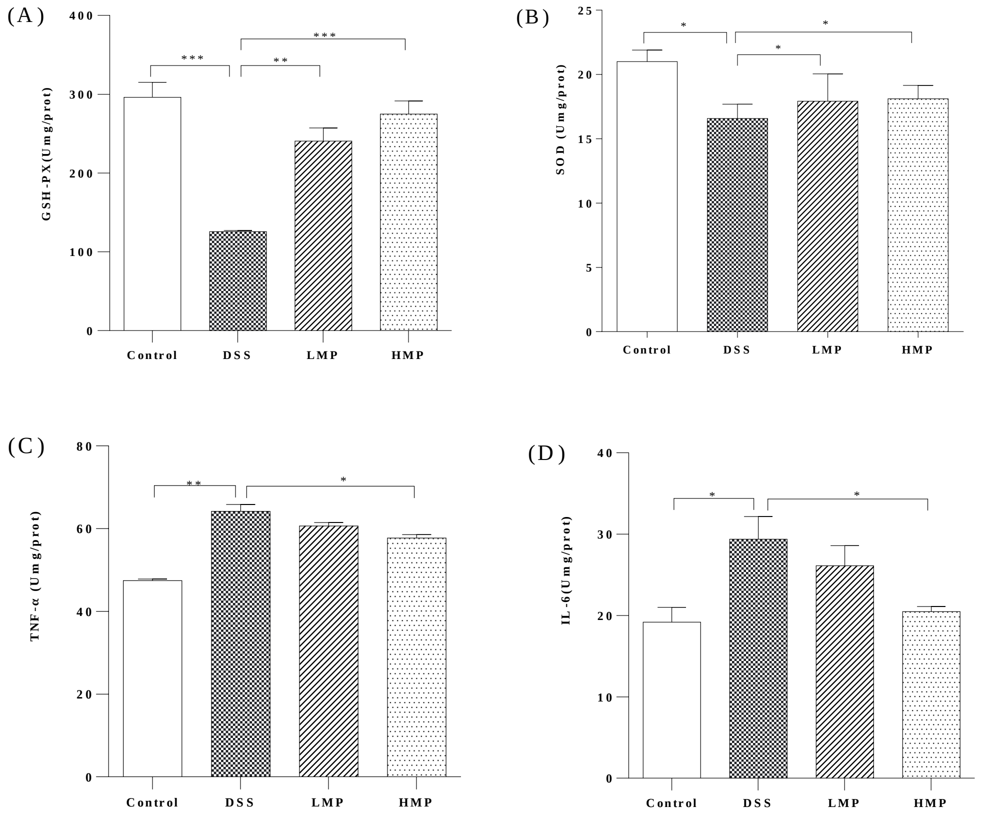

Scheme 2. Effect of MP on serum oxidative stress (GSH-Px (**A**) and SOD (**B**)), and inflammatory response (TNF-α (**C**) and IL-6 (**D**)) in DSS-induced colitis mice. The data are presented as means ± SEM. Significant differences in the results were analyzed by 1-way ANOVA analysis with post hoc independent samples *t*-tests (p values <0.05, <0.01, and <0.001 are indicated by *, **, and ***, respectively).
